# Isavuconazole: A Promising Salvage Therapy for Invasive Mucormycosis

**DOI:** 10.7759/cureus.2547

**Published:** 2018-04-29

**Authors:** Muhammad Shafiq, Zafar Ali, Rehman Ukani, Joseph Brewer

**Affiliations:** 1 Department of Internal Medicine, University of Missouri Kansas City (UMKC); 2 Department Chair - Infectious Disease, Saint Luke's hospital , Plaza , Kansas City , Missouri.

**Keywords:** invasive mucormycosis, isavuconazole, salvage therapy, amphotericin b

## Abstract

A patient with invasive mucormycosis whose disease progresses despite optimal treatment including surgical debridement, intravenous (IV) amphotericin B, and control of the predisposing factors can be clinically challenging. We report a case of a 67-year-old Caucasian man with invasive mucormycosis that did not respond to first-line treatment. He was subsequently started on isavuconazole in addition to amphotericin B. The patient’s disease progression stopped; he then received IV amphotericin B for 50 days and isavuconazole for four months. Repeated magnetic resonance imaging (MRI) of the orbit and face nine months later, while off the antifungal medications, showed stable disease. This outcome is promising for patients with invasive mucormycosis who are either intolerant to amphotericin B or do not respond favorably to it.

## Introduction

Mucormycosis predominantly affects patients with underlying comorbidities such as immunosuppression or diabetes mellitus. Early recognition and timely management are the keys to successful treatment. Amphotericin B and its lipid formulations are considered first-line antifungal agents in the treatment of mucormycosis [1-2]. However, when the patient’s invasive disease progresses despite optimal treatment with surgical debridement, intravenous (IV) amphotericin B, and control of the predisposing factors, this presents a clinical challenge as there are no good data available yet to suggest the next step.

## Case presentation

A 67-year-old Caucasian man with a significant history of recently diagnosed type 2 diabetes mellitus (T2DM) and essential hypertension presented to the hospital with chief concerns of diplopia with an extreme gaze, right eye pain, and sinus congestion for about two weeks.

**Figure 1:**
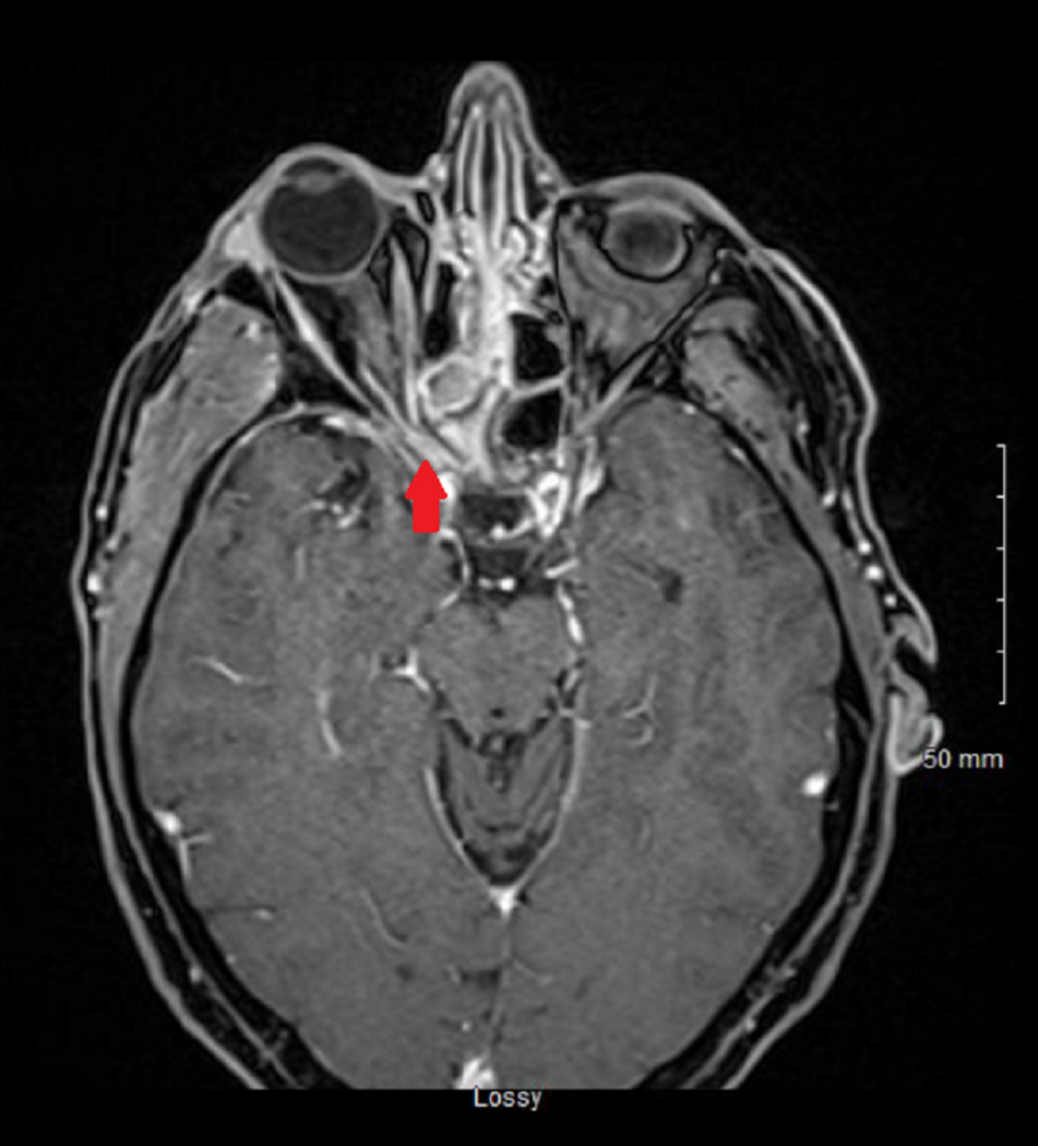
Magnetic resonance image of the axial section of the orbits and face Right eye proptosis, right-sided sinuses involvement, and compression of the right optic nerve (red arrow) can be seen.

At the time of admission, the patient was afebrile, had a blood pressure of 160/67 mmHg and pulse of 64/minute but had a white blood cell count of 14,540/µL. The patient’s blood glucose was 469 mg/dL, anion gap levels were within reference range, and his glycosylated hemoglobin (HbA1c) was 12.4%. Computed tomography (CT) and magnetic resonance imaging (MRI) of the orbit and face revealed severe sinusitis with possible orbital cellulitis and optic nerve compression (Figure 1).

On the day of admission, the patient was started on an IV ampicillin and sulbactam combination (3000 mg/mL every six hours) and IV vancomycin (1500 mg/mL loading dose; 1250 mg/mL every 12 hours maintenance dose; target vancomycin trough of 10 to 20 mg/mL due to the severity of the infection). The patient was seen by the infectious disease (ID) team on day two of admission. The ID team recommended continuing vancomycin, switched the ampicillin/sulbactam combination medication to piperacillin/tazobactam (3375 mg/mL every six hours) and started the patient on empiric IV liposomal amphotericin B (400 mg/mL daily) given the concerns for invasive fungal infection. The patient was seen by ophthalmology team, and they recommended no acute surgical intervention. However, the otorhinolaryngology (ENT) team performed an endoscopy of the nasal sinuses on the second hospital day, and the patient required extensive debridement of the necrotic tissue of the right sinuses.

Biopsy results from the nasal sinuses showed broad hyphae with infrequent septations, haphazard branching, and numerous bizarre forms. These morphologic features were consistent with mucormycosis. Given this finding, the patient was continued on IV liposomal amphotericin B.

**Figure 2:**
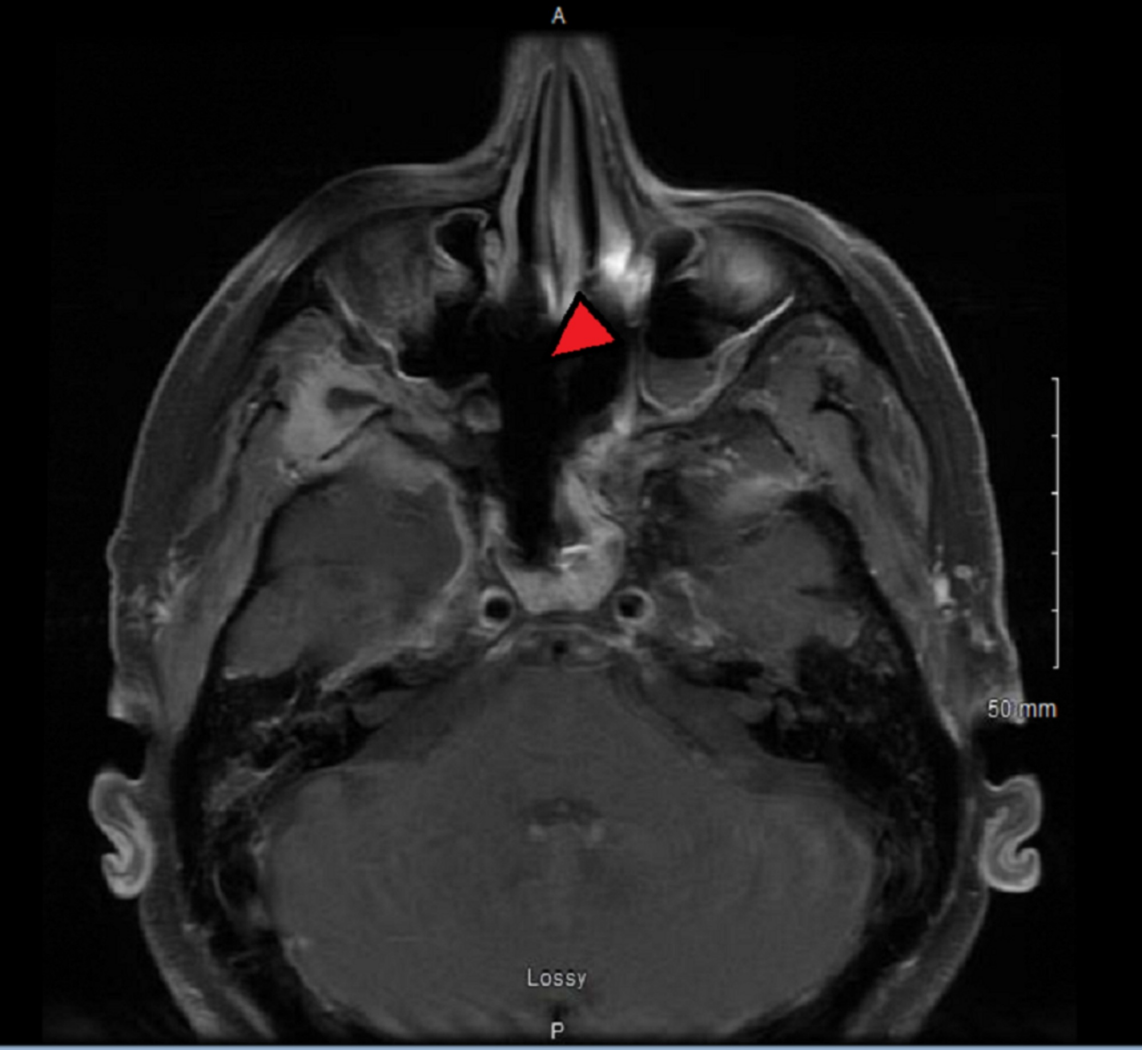
Magnetic resonance image of the axial section of the orbits and face Destruction of the right-side maxillary sinus can be seen (red arrow).

A second MRI of the orbit and face was repeated on the fourth hospital day and showed the progression of the disease including involvement of right retrobulbar fat and right optic nerve, persistent non-enhancing, and likely necrotic tissue extending from the right pterygopalatine fossa into the right masticator space (Figure 2). Suspected osteomyelitis was noted at the base of the skull, and we noted asymmetric right frontotemporal pachymeningeal enhancement with wispy leptomeningeal enhancement along the middle cranial fossa, which was suggestive of meningitis.

The neurosurgery team was consulted as the patient had meningeal spread as well. The neurosurgery team deemed the patient inoperable.

Given the progression of the disease despite the standard-of-care treatment (i.e., surgical debridement, IV amphotericin B, and control of blood glucose), a literature search was done to find the next best step. Isavuconazole has been used, according to a few case reports [3-5], as salvage treatment in patients whose condition either failed the IV amphotericin B or were intolerant to it. Therefore, our patient was started on IV isavuconazole (372 mg/mL every eight hours) on the fifth hospital day, and IV amphotericin B (400 mg/mL daily) was continued. Since patient tissue aerobic and anaerobic cultures as well as blood cultures remained negative, piperacillin/tazobactam and vancomycin were discontinued on the fifth hospital day.

An MRI of the orbit and face was repeated 48 hours after the patient was started on isavuconazole and revealed a stable disease. The patient’s IV isavuconazole dose was reduced to 372 mg/mL daily (after receiving IV isavuconazole every eight hours for 48 hours). An MRI was obtained one week later, and it also showed stable disease with no further progression. The patient was switched to oral isavuconazole (372 mg daily), and IV amphotericin B (400 mg/mL daily) was continued. As the patient continued to improve, he was ultimately discharged after 19 hospital days on this regimen with a plan for outpatient follow-up evaluations with the ID, ENT, and ophthalmology teams. The patient was blind in his right eye with cranial nerves III, IV and VI palsies at the time of discharge. However, his left eye was completely intact with normal vision and movement.

**Figure 3:**
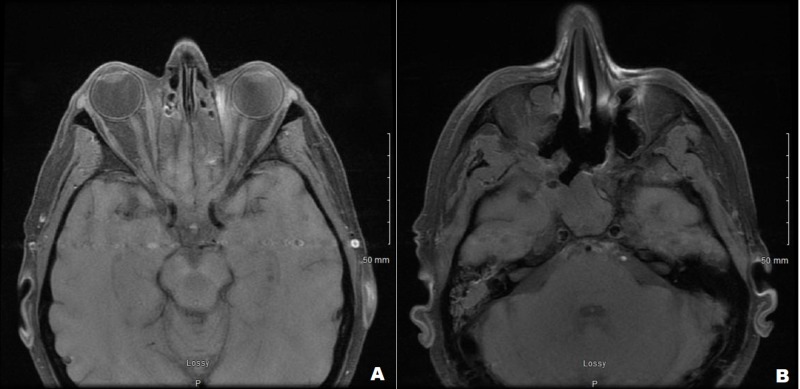
Magnetic resonance image of the axial section of the orbits and face Right eye proptosis has improved and right-sided sinuses involvement remains stable.

During outpatient care, he continued to receive IV amphotericin B (400 mg/mL daily) and oral isavuconazole (372 mg daily). An MRI of the orbit and face after five weeks of this therapy showed some interval improvement of the infectious process (Figure 3). After receiving IV amphotericin B (400 mg/mL daily) for 50 days, the medication was stopped, and the patient was continued on oral isavuconazole (372 mg daily). The patient's renal function was checked weekly as the patient was on IV amphotericin B. The patient had baseline creatinine of 1.0 mg/dL, it peaked at 1.8 mg/dL and returned to 1.4 mg/dL once the IV amphotericin B was stopped. Fungal cultures and blood samples were monitored for six weeks, and they remained negative.

An MRI of the orbit and face was obtained two months later while the patient was treated with oral isavuconazole (372 mg daily), and the imaging continued to show stable disease. Therefore, oral isavuconazole was stopped. By this time, the patient had isavuconazole for a total of four months (both via IV and oral doses). Besides headaches, the patient tolerated the isavuconazole well without any other significant side effects.

An MRI of the orbit and face was obtained again nine months after stopping oral isavuconazole, and his disease process has remained stable. Imaging at this time showed some improvement in the condition. The ID team stated the patient was cured. The ENT team still monitors the patient periodically, and, as of this writing, the patient has been over one year off of antifungal medications.

Regarding the patient’s diagnosis of uncontrolled T2DM, the patient was initially started on basal insulin while as an inpatient, but he was discharged on oral antihyperglycemic agents (metformin, 500 mg twice daily with meals and sitagliptin, 100 mg daily) given better control with oral agents at the time of discharge. His HbA1c improved to 5.0% from 12.4% in three months. The patient’s metformin has been stopped, and his sitagliptin dose has been reduced to 50 mg daily and is the only diabetic medication for the patient as of this writing.

## Discussion

No good data exists yet for the appropriate next steps in treating invasive or disseminated mucormycosis that has continued to progress despite surgical debridement, IV amphotericin B, and control of predisposing factors. Our case report represents a life-threatening invasive rhinocerebral mucormycosis that failed the conventional first-line treatment. However, when isavuconazole was used as salvage therapy, our patient’s disease did not progress further. Rather, radiography images suggest the condition has improved. This is a promising outcome for patients with invasive or disseminated mucormycosis who are intolerant to amphotericin B or their condition does not respond to it. A few other case reports have supported this as well [3-5].

Isavuconazole is a new triazole that was recently compared with amphotericin B in a single arm, open-label trial that found similar efficacy if isavuconazole is used as the first-line agent [6]. However, no randomized trials are yet available to suggest the superiority of one anti-fungal agent over another. The common side effects related to isavuconazole are nausea, vomiting, diarrhea, headaches, fatigue, insomnia, peripheral edema, hypokalemia, and occasionally dyspnea. Besides a headache, our patient didn’t experience any other side effects.

Delaying the anti-fungal therapy by more than six days can double the mortality associated with mucormycosis by the end of 12 weeks [2]. Therefore, early initiation of anti-fungal drugs, surgical debridement of the necrotic tissue as soon as the diagnosis is confirmed, and reversal of the predisposing factors are the key elements in overall management.

Mucormycosis predominantly affects patients who are immunocompromised or have diabetes [7]. Clinical presentation depends largely on the site of involvement. Rhinocerebral mucormycosis is the most common form of this infection which is thought to occur after inhalation of spores into the paranasal sinuses. It mainly affects patients with diabetes, and one study reported 70% of patients with rhino-cerebral mucormycosis had diabetes and many had ketoacidosis at the time of presentation [8]. Other sites that can be involved include lungs, skin, gastrointestinal tract, or it can present as a disseminated infection [9-10].

## Conclusions

If a patient fails to respond to standard first-line management for invasive mucormycosis which includes early initiation of IV amphotericin B, surgical debridement of the necrotic tissue as soon as the diagnosis is confirmed, and reversal of the predisposing factors, isavuconazole is a very reasonable option as salvage therapy in such scenarios.
